# Elevated adipose tissue associated IL-2 expression in obesity correlates with metabolic inflammation and insulin resistance

**DOI:** 10.1038/s41598-020-73347-y

**Published:** 2020-10-01

**Authors:** Shihab Kochumon, Ashraf Al Madhoun, Fatema Al-Rashed, Reeby Thomas, Sardar Sindhu, Ebaa Al-Ozairi, Fahd Al-Mulla, Rasheed Ahmad

**Affiliations:** 1 Immunology and Microbiology Department, Dasman Diabetes Institute, Jasim Mohamad Al Bahar St., P.O. Box 1180, 15462 Kuwait City, Kuwait; 2 Animal and Imaging Core Facilities, Dasman Diabetes Institute, Kuwait City, Kuwait; 3 Genetics and Bioinformatics, Dasman Diabetes Institute, Kuwait City, Kuwait; 4 Immunology Department, Ministry of Health, Kuwait City, Kuwait; 5 Medical Division, Dasman Diabetes Institute, Kuwait City, Kuwait

**Keywords:** Immunology, Biomarkers

## Abstract

Adipose tissue (AT) associated cytokines are involved in the development of chronic low-grade inflammation in obese individuals. IL-2, a pleiotropic cytokine, contributes to immune alterations during inflammation. However, the interaction between AT-IL-2 and other inflammatory biomolecules in obesity remains elusive. We investigated whether AT-IL-2 expression was associated with markers of inflammation and insulin resistance in overweight/obese individuals. Subcutaneous fat tissues were collected from 56 individuals (lean/overweight/obese) for RNA extraction. IL-2 and inflammatory mediators were quantified by qRT-PCR and immunohistochemistry. CRP was measured by ELISA. AT-IL-2 expression was higher in obese compared with lean individuals (P < 0.021) and correlated with BMI. IL-2 correlated with interleukins IL-8 and IL-12A (r = 0.333–0.481; *p* = 0.0001–0.029); as well as with chemokines and their receptors including CCL5, CCL19, CCR2 and CCR5 (r = 0.538–0.677; p < 0.0001). Moreover, IL-2 correlated with toll-like receptors (TLR2, TLR8, TLR10), interferon regulatory factor 5 (IRF5) and cluster of differentiation CD11c (r = 0.282–0.357; *p* < 0.039). Notably, IL-2 was associated positively with fasting blood glucose (FBG), HbA1c, TGL and CRP (r ≥ 0.423;P ≤ 0.007). In multiple regression analysis, IL-2 is an independent predictor of IL-8, IL-12A, TLR10, TGL and HbA1c. Overall, our data demonstrate that increased expression of the AT-IL-2, in obesity, may represent a novel biomarker for progression of metabolic inflammation and insulin-resistance.

## Introduction

The prevalence of obesity is increasing dramatically, and nearly two third of the of global population is either overweight or obese^1, 2^. Genetic and environmental factors cause substantial variability in the onset of metabolic complications for any given degree of obesity^3–6^. In obese subjects, the pathophysiology related to adverse metabolic profile is due to alterations in several factors such as fat distribution, glucose homeostasis, dyslipidemia, and high blood pressure; yet, chronic systemic low-grade inflammation is one of the most consistent factor involved^7, 8^. Interestingly, the precise mechanisms related to inflammation and metabolic syndrome are still unclear^9, 10^.

Adipose tissue (AT) is a plastic, dynamic and endocrine organ. As a primary fat and triacylglycerol depot, AT has the plasticity to adjust to a surplus in energy through progressive adipocyte hypertrophy as well as proliferation and differentiation of pre-adipocyte precursor cells as the mechanisms that are associated with active neovascularization and extracellular matrix remodeling. Furthermore, AT is an active endocrine organ that produces and secretes hormones, adipocytokines and chemokines^11^. Together, aberrant AT vascularization is associated with the inflammatory response and metabolic dysfunctions observed in obese individuals^12, 13^.

Several tissue immunometabolic studies have investigated the impact of intracellular signaling alterations within the immune cells in tissues and the changes in systematic metabolism due to different health conditions^14, 15^. Several proinflammatory markers have been reported to be consistently correlated with the obesity levels and associated complications including insulin resistance and cardiovascular diseases^16, 17^. In obese AT, activation of inflammatory mediators induces the release of free fatty acids from triglyceride (TG) depots during lipolysis, which in turn, promote the infiltration and activation of circulating leukocytes. The various leukocyte populations produce and secrete cell type-specific inflammatory mediators that play a key role in the crosstalk among heterogeneous effector cell populations within the obese AT^18, 19^.

Here, we will focus on IL-2 which is a monomeric glycoprotein that is primarily secreted from the circulating activated CD4^+^ and CD8^+^ T cells, and to a lesser extent, by dendritic cells and macrophages^20, 21^ as well as invariant natural killer T (iNKT) cells^22^. IL-2 exerts pleiotropic effects on the immune system and target tissues. It plays a crucial role in regulating immune cell proliferation, activation and homoeostasis; well-reviewed elsewhere^23, 24^. However, IL-2 expression and its relationship with other significant markers of metabolic inflammation in obesity is not well understood. Herein, we report that adipose tissue IL-2 gene/protein expression was upregulated in obese individuals compared to lean individuals. Further, this elevation of IL-2 is associated with insulin resistance as well as with several important inflammatory markers. Importantly, in our obese cohort, two diverse clusters of IL-2 gene expression were identified and in those with high expression, IL-2 gene expression correlated positively with fasting blood glucose (FBG) and glycated hemoglobin (Hb1Ac).

## Results

### Demographic and clinical characteristics of study population

The physiological and biochemical characteristics of the 56 individuals included in this study are detailed in Table 1. All participants were below 60 years old; age ranged between 33 to 57 years, with no significant differences in height. Relative to lean individuals, overweight and obese individuals showed significantly increased BMI, waist circumference, total body fat and plasma triglycerides (Table 1). Whereas, mean values for total plasma cholesterol and LDL levels were comparable between three groups, HDL was significantly higher in lean individuals (Table 1). In this study, patients with diabetes were excluded. However, fasting blood glucose and HbA1c measurements ranged between 4.3–6.1 mmol/L and 5.0–6.3%, respectively, suggesting that the population included normal and/or pre-diabetics. This was further supported by a statistically significant increase in HOMA-IR in obese patients relative to lean individuals (Table 1).

**Table 1:** Demographic and clinical characteristics study population.

Phenotype	Lean	Overweight	Obese	Lean vs overweight	Lean vs obese
	(*n* = 9) (mean ± SD)	(n = 18) (mean ± SD)	(*n* = 29) (mean ± SD)	(P-value)	(P-value)
Age (years)	41.8 ± 8.1	43.3 ± 11.5	44.7 ± 12.9	0.730	0.529
Weight (kg)	63.0 ± 12.6	80.2 ± 9.4	94.7 ± 14.2	0.0004	< 0.0001
Height (cm)	1.7 ± 0.1	1.7 ± 0.1	1.65 ± 0.11	0.766	0.657
BMI (kg/m^2^)	22.6 ± 2.4	28.4 ± 1.1	34.90 ± 3.30	< 0.0001	< 0.0001
Waist (cm)	81.9 ± 13.2	95.7 ± 8.8	107.16 ± 13.04	0.005	< 0.0001
Body fat (%)	27.3 ± 5.8	32.3 ± 5.0	39.61 ± 4.21	0.037	< 0.0001
FBG (mmol/L)	5.0 ± 0.7	5.3 ± 0.7	5.6 ± 0.8	0.258	0.160
TGL (mmol/L)	0.6 ± 0.2	1.2 ± 0.6	1.4 ± 0.9	0.001	0.0001
Chol (mmol/L)	5.3 ± 1.2	5.0 ± 0.7	5.0 ± 1.1	0.351	0.482
HDL (mmol/L)	1.6 ± 0.5	1.2 ± 0.2	1.2 ± 0.3	0.038	0.001
LDL (mmol/L)	3.4 ± 1.0	3.2 ± 0.7	3.2 ± 1.0	0.582	0.705
HbA1c (%)	5.7 ± 0.5	5.5 ± 0.5	5.7 ± 0.7	0.344	0.952
Insulin (mU/L)	9.4 ± 7.6	6.0 ± 2.2	13.9 ± 10.0	0.769	0.157
HOMA-IR	1.4 ± 0.7	2.1 ± 1.9	4.1 ± 3.8	0.312	0.020
WBC	5.5 ± 1.7	6.2 ± 1.6	6.5 ± 2.0	0.282	0.173

### Adipose tissue IL-2 gene expression is associated with obesity

**Figure 1:**
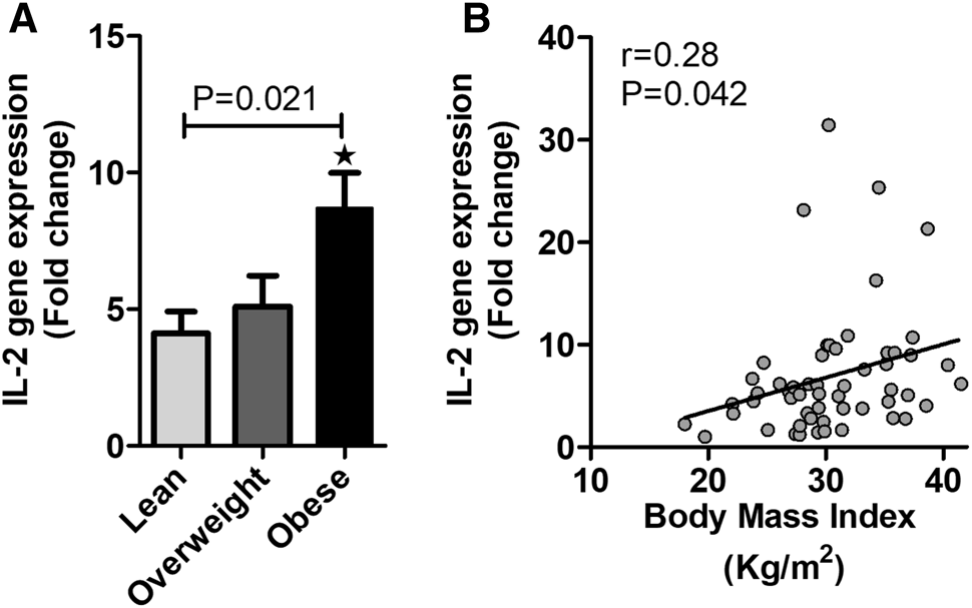
Increased adipose tissue associated IL-2 gene expression in obese individual. **(A**) qRT-PCR analysis for IL-2 RNA isolated from adipose tissues from lean, overweight and obese individuals. (**B)** In the studied population, IL-2 transcript levels, in adipose tissue, are positively associated BMI.

In humans, plasma levels of IL-2 and their relationship with BMI and obesity status remain controversial^25–27^. Knowing that IL-2 gene expression is modulated in various inflammatory disorders and that it is expressed and secreted by AT- resident iNKT cells^22^, we hypothesized a differential expression of IL-2 gene in AT biopsies from individuals with different BMI. Indeed, relative to levels of IL-2 transcripts observed in RNA extracts from subcutaneous AT obtained from lean individuals, qRT-PCR analysis revealed a surge of the IL-2 transcripts in overweight participants and a statistically significant increase in its levels in obese individuals (*p* = 0.021) (Fig. 1A). Notably, IL-2 expression exhibited a substantial positive correlation with the plasma BMI (r = 0.280,* P* = 0.042; Fig. 1B).

**Figure 2:**
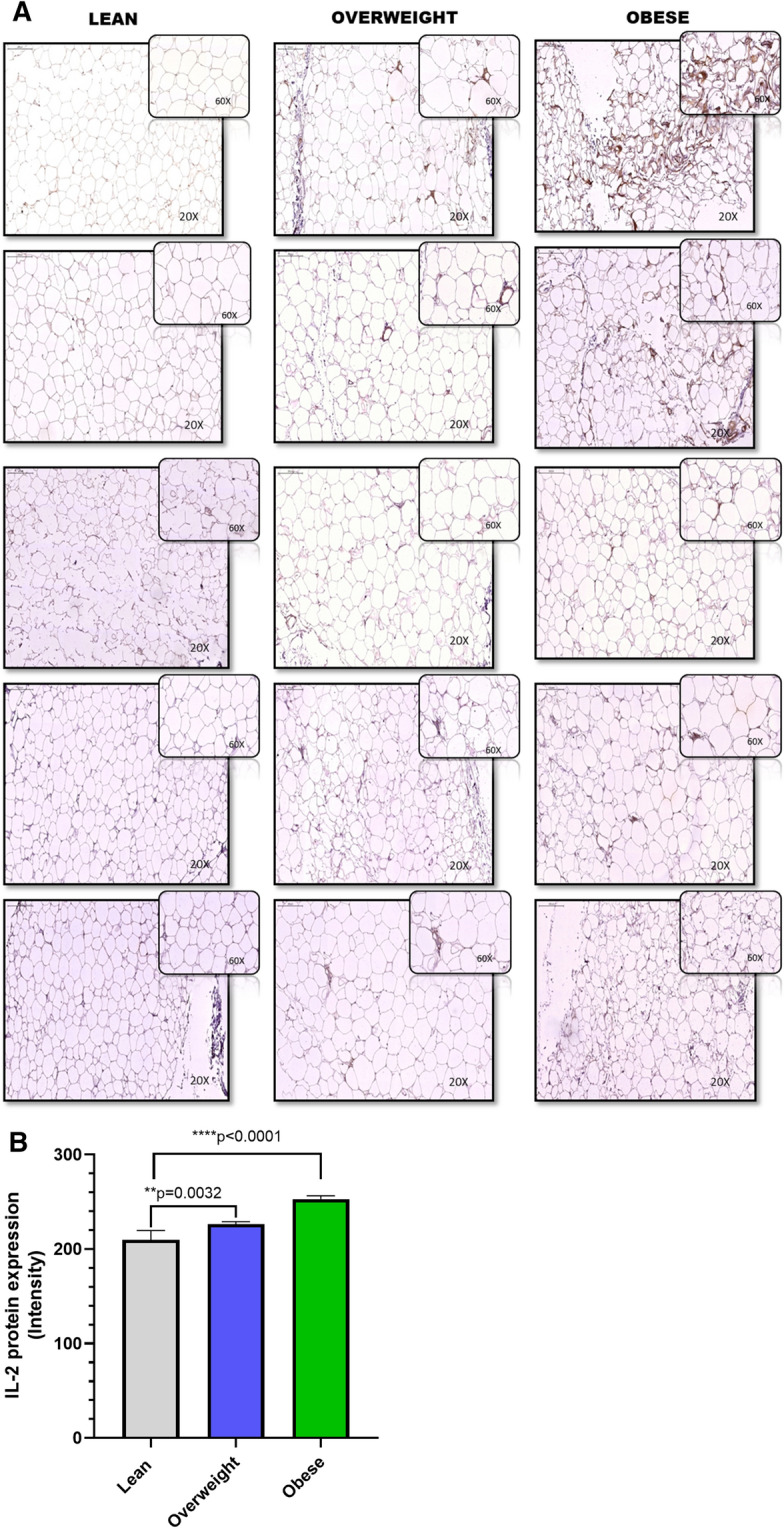
Increased adipose IL-2 protein expression in obese individuals. IL-2 protein expression was determined in five lean, 5 overweight, and 5 obese non-diabetic individuals using immunohistochemistry (IHC) as described in “Material and methods”. (**A**) The representative IHC images obtained from three independent determinations with similar results show increased adipose tissue IL-2 expression (× 20 or insert × 60 magnification) in overweight and obese individuals compared with lean. (**B**) Staining intensity was determined based on Aperio-positive pixel counts (Aperio software algorithm version 9.0) URL: https://www.3dhistech.com/products-and-software/hardware/pannoramic-digital-slide-scanners/pannoramic-scan-2/). IL-2 protein was expressed as IHC intensity in arbitrary units and presented as mean ± SEM. The asterisks ** and **** represent significance levels of P < 0.004, and P < 0.0001, respectively.

Consistent with IL-2 mRNA data, the immunohistochemistry (IHC) data revealed that IL-2 protein levels were significantly (*P* = 0.0032 and *P* = 0.0001, respectively) higher in individuals with obesity as compared to lean controls (Fig. 2A,B).

### In adipose tissue, increased IL-2 gene expression relates with inflammatory signatures

**Figure 3:**
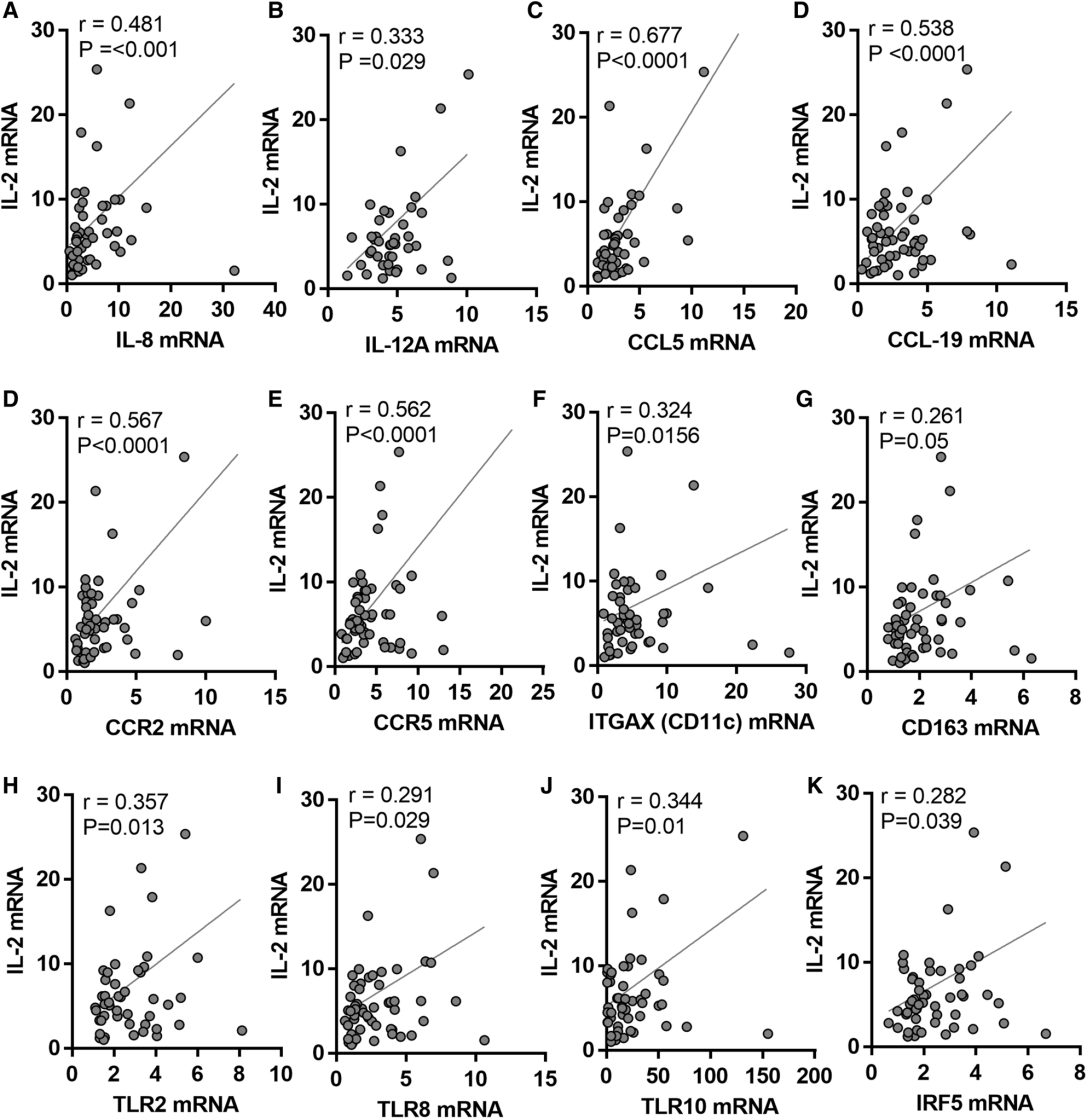
Increased adipose tissue associated IL-2 gene expression is correlated with inflammatory markers. Subcutaneous adipose tissues were obtained from lean, overweight, obese individuals. mRNA expression of IL-2, IL-12A, CCL5, CCL19, CCR2, CCR5, CD11c, CD163, TLR2, TLR8, TLR10, and IRF5 was detected by real-time RT-PCR and represented as fold change over controls. (**A–K)** In the studied population, IL-2 transcript levels, in adipose tissue, are positively correlated with inflammatory markers.

IL-2 is a potent proinflammatory Th1 cytokine and it mediates T cells activation, yet, its crosstalk with other chemokines/cytokines in the AT is not fully clarified. We were interested in knowing whether the elevated IL-2 mRNA expression, in overweight and obese AT, was concordant with the expression of inflammatory markers. As described in Table 2 and Fig. 3, IL-2 transcript levels displayed a statistically significant positive correlation with proinflammatory cytokines including IL-8 (r = 0.481; *p* = 0.001) and IL-12A (r = 0.333; *p* < 0.05); and a moderate correlation with IL-1β (r = 0.268; *p* = 0.082).

**Table 2:** Correlation of IL-2 with various inflammatory markers in non‐diabetic overweight and obese individuals.

Inflammatory marker	Pearson Correlation	
	r value	*P*-value
IL-1β	0.268	0.082
IL-5	− 0.105	0.454
IL-6	0.01	0.943
**IL-8**	0.481**	** < 0.0001**
IL-10	0.064	0.642
**IL-12A**	0.333*	**0.029**
IL-13	− 0.11	0.437
IL-18	0.074	0.595
CCL2	− 0.069	0.619
CCL3	0.135	0.337
**CCL5**	0.677**	** < 0.0001**
CCL8	− 0.086	0.558
CCL11	0.01	0.944
CCL15	− 0.167	0.21
**CCL19**	0.538**	** < 0.0001**
CCL20	0.201	0.133
CCR1	− 0.049	0.724
**CCR2**	0.567**	** < 0.0001**
**CCR5**	0.562**	** < 0.0001**
CD16	0.076	0.577
**ITGAX (CD11c)**	0.324*	**0.0156**
**CD163**	0.261*	**0.05**
TNFα	0.14	0.317
TGFβ	0.077	0.579
IFNB1	0.052	0.702

Bold is used to highlight the significant values

We then extended the correlation analysis at transcriptional level to include members of the C–C motif chemokines (Table 2). Out of eight members examined, IL-2 mRNA levels correlated significantly with those of CCL5 (r = 0.677; *p* < 0.0001) and CCL19 (r = 0.538; *p* < 0.0001) as well as chemokine receptors CCR2 (r = 0.567; *p* < 0.0001) and CCR5 (r = 0.562; *p* < 0.0001). Further, IL-2 transcripts also associated in tandem with M1 macrophage markers CD163 (r = 0.261; *p* = 0.05) and CD11c (ITGAX) (r = 0.324; *p* = 0.0156) (Table 2, Fig. 3). However, no correlation between the transcript levels of IL-2 and transforming growth factor beta (TGFβ), tumor necrosis factor alpha (TNFα), or interferon beta-1 (IFNB1) was detected in the study population. In order to determine which inflammatory parameters independently correlated with IL-2, the parameters having significant association with IL-2 were included for further multiple stepwise regression analysis. Multiple linear regression analysis indicated that the gene expression of IL-8 and IL-12A was independently associated with that of IL-2 (Table 3).

**Table 3:** Multiple linear regression analysis between IL-2 and other markers in non-diabetic overweight/obese individuals, dependent variable: IL-2.

ANOVA (sig) R2 = 0.575; *p* < 0.0001		
Predictor variable	β-value	*P-*value
Triglyceride	3.883	0.001
IL-8	0.317	0.009
IL-12A	1.245	0.004

### Association between AT IL-2 expression and toll-like receptors (TLRs)

The TLRs are important component of the innate immune system and play crucial role in inflammation via triggering the expression of cytokines and chemokines including IL-2. Thus, we expected to find a positive correlation between IL-2 expression and TLRs in the AT in obese individuals. Notably, a statistically significant positive correlation was detected between IL-2 transcripts and that of TLR-2, -8 and -10 (r = 0.291; *p* < 0.029; Table 4). We did not find association between mRNA expression levels of IL-2 and those of TLR3, -4, -7 or -9. Further, this positive association was extended to include the downstream effectors of the TLR signaling pathways, and a positive correlation was found between gene expression of IL-2 and IRF5 (r = 0.282; *p* = 0.039). In addition, adipose tissue protein expression was determined regarding IL-8, CCL5, CD11c, TLR10 and IRF5 in biopsies from lean, overweight and lean individuals (Supplementary Figure S1A) and their correlation was shown with IL-2 protein expression in the respective adipose tissue samples (Supplementary Figure S1B).

**Table 4:** Correlation of IL-2 with toll-like receptors and associated singling molecules in non‐diabetic overweight/obese individuals.

TLRs/signaling molecules	Pearson correlation	
	r value	*P-*value
**TLR2**	0.357*	**0.013**
TLR3	− 0.036	0.803
TLR4	− 0.003	0.982
TLR7	0.174	0.193
**TLR8**	0.291*	**0.029**
TLR9	0.049	0.719
**TLR10**	0.344*	**0.01**
MyD88	0.171	0.203
IRAK1	0.141	0.304
TRAF6	0.024	0.862
**IRF5**	0.282*	**0.039**

Bold is used to highlight the significant values

### IL-2 gene expression in obese individuals shows two clusters

**Figure 4:**
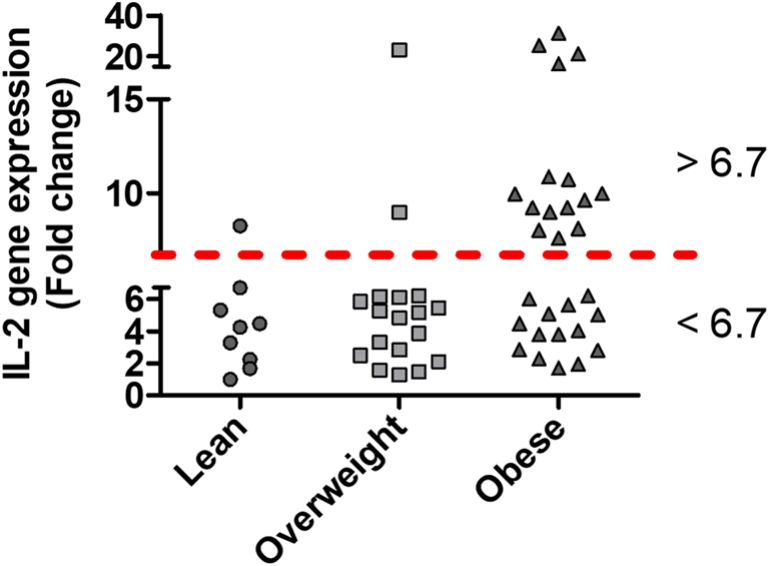
AT associated IL-2 gene expression in obese individuals exist in two distinct expression clusters. qRT-PCR analysis was performed for IL-2 mRNA isolated from adipose tissues from lean, overweight and obese individuals. IL-2 gene expression in obese individuals displayed two distinct clusters high (> 6.7-fold change) and low (< 6.7-fold change).

Further investigating the IL-2 gene expression in obese individuals, we observed a distinct, two clusters segregation, above and below the threshold of 6.7-fold changes (Fig. 4); accordingly, we decided to further analyze each cluster/group independently.

Interestingly, the two clusters revealed a differential pattern of correlations between IL-2 mRNA expression and different inflammatory markers in the AT (Table 5). In obese individuals having high IL-2 (> 6.7-fold changes), gene expression levels displayed a significant positive association with the expression levels of the studied inflammatory factors (Table 5), including IL-8, CCL15, CCL19, CD16 and TLR10 (r values ranged between 0.572 to 0.879; *p* = 0.0001 to 0.026; Table 5); and to a less extent with that of CCR2, CCR5, IRF5 and MyD88 (r ≥ 0.451; and *p* ranged between 0.069 to 0.090). On the other hand, correlation analysis of obese group expressing IL-2 < 6.7-fold changes revealed non-significant negative correlations with all studied inflammatory markers except for that of IL-8 and CCL11. In the later group, no associations were noticed between IL-2 and CCL2, CCL20 or MyD88 transcripts (Table 5). In addition, partial correlation analysis of IL-2 with other markers was carried out after adjusting for high/low IL-2 cluster variances (Supplementary Table S1).

**Table 5:** Correlation of IL-2 low and high expression clusters observed in obese individuals with various cytokines/chemokines and TLR.

Inflammatory marker	Pearson Correlation			
	IL-2 < 6.7-folds		IL-2 > 6.7-folds	
	(r value, *n* = 14)	*P-*value	(r value, *n* = 15)	*P-*value
IL-1B	0.051	0.876	0.229	0.451
IL-5	0.011	0.969	− 0.281	0.311
IL-6	0.154	0.600	0.096	0.744
IL-8	0.309	0.354	0.635*	**0.015**
IL-10	− 0.027	0.934	0.265	0.339
IL-12A	− 0.022	0.950	0.473	0.142
IL-13	− 0.301	0.342	0.057	0.847
IL-18	− 0.182	0.593	0.049	0.873
CCL2	− 0.004	0.990	− 0.082	0.773
CCL3	− 0.182	0.593	0.049	0.873
CCL5	− 0.165	0.627	0.217	0.522
CCL8	0.240	0.453	0.004	0.989
CCL11	0.388	0.19	− 0.333	0.244
CCL15	− 0.162	0.579	0.736**	**0.002**
CCL19	− 0.115	0.707	0.879**	** < 0.0001**
CCL20	0.007	0.98	0.13	0.644
CCR1	− 0.377	0.227	0.088	0.765
CCR2	0.093	0.785	0.503	0.067
CCR5	− 0.096	0.743	0.451	0.091
CD16	− 0.09	0.759	0.621*	**0.018**
ITGAX	− 0.141	0.645	0.396	0.160
CD163	− 0.042	0.887	0.334	0.243
TNFα	− 0.248	0.392	− 0.056	0.85
TGFβ	− 0.152	0.605	− 0.162	0.597
IFNB1	0.237	0.435	0.215	0.442
TLR10	− 0.457	0.116	0.572*	**0.026**
IRF5	− 0.041	0.889	0.466	0.08
MyD88	0.01	0.974	0.453	0.09

Bold is used to highlight the significant values

### Association between AT IL-2 expression and clinical metabolic parameters

**Figure 5:**
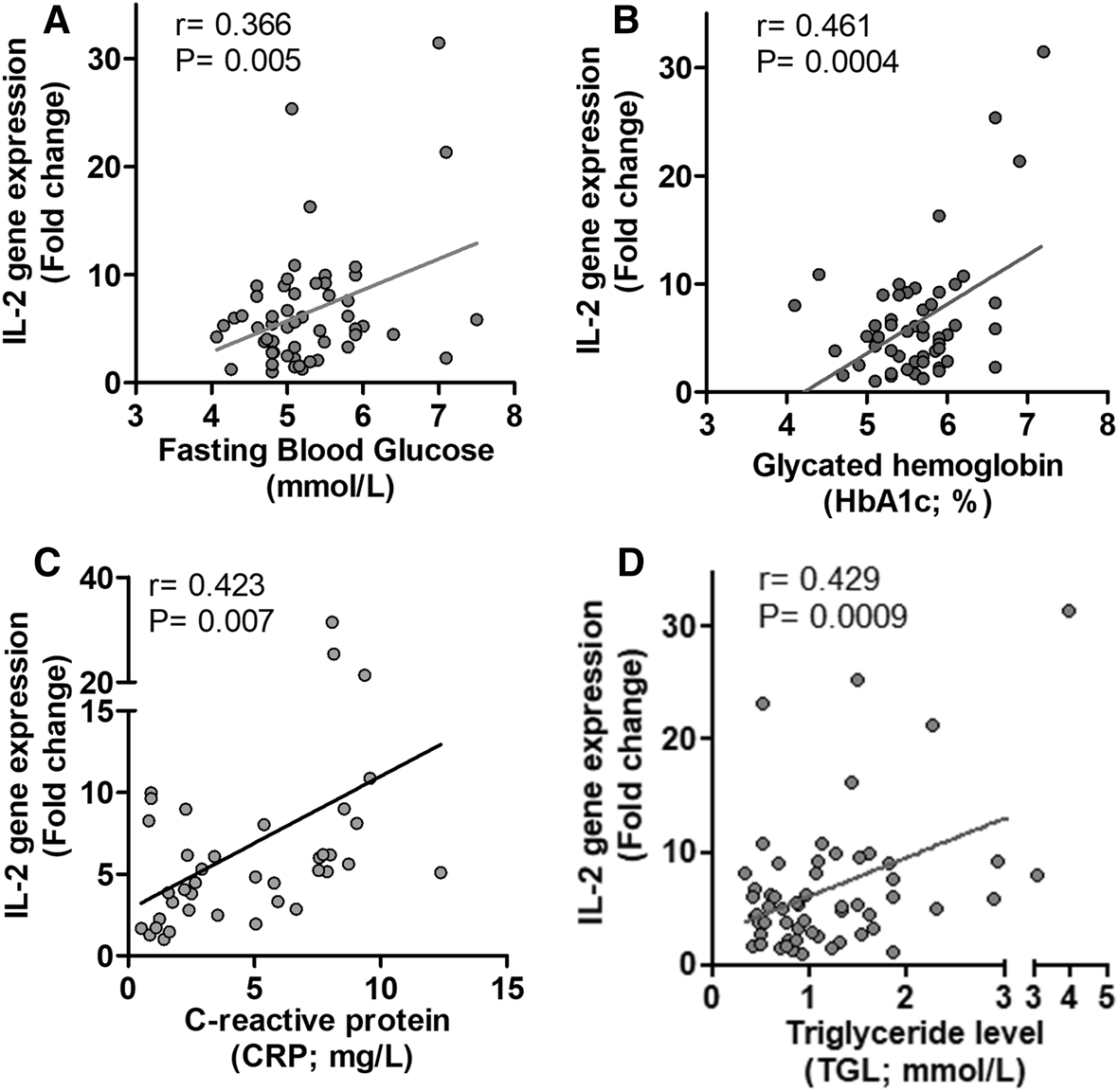
Correlation of IL-2 gene expression with clinical parameters. (**A–D**) IL-2 RNA expression levels, in AT, exhibited a substantial positive correlation with the plasma fasting blood glucose (FBG), glycated hemoglobin (HbA1c), C-reactive protein (CRP), and triglyceride levels (TGL).

Our data indicate that IL-2 positively associates with selective inflammatory markers. Next, we asked whether the changes in adipose tissue IL-2 gene expression associated with clinico‐metabolic signatures. As shown in Fig. 5A–D, IL-2 expression levels were found to be associated positively with that of plasma FBG (r = 0.366; *p* = 0.005), HbA1c (r = 0.461;* p* = 0.0004), CRP (r = 0.423;* p* = 0.007), and TGL (r = 0.429;* p* = 0.0009). However, IL-2 did not correlate with the lipid profile markers (HDL, LDL and total cholesterol). Notably, TGL independently associated with IL-2 as determined by multiple linear regression analysis.

Since we found two clusters of IL-2 gene expression in obese individuals, we asked whether the changes in AT—high and -low IL-2 gene expression clusters were associated with clinico-metabolic signatures. To this end as shown in Table 6, the two clusters revealed variable associations with several clinical markers. However, noticeable differences were observed in relation to insulin resistance markers. In obese individuals with high AT expression of IL-2, > 6.7-fold changes; both FBG (r = 0.566; *p* = 0.028) and HbA1c (r = 0.653; *p* = 0.008) show a strong positive correlation with IL-2 transcript levels in the AT (Table 6). Thus, obese individuals with high levels of IL-2 might be at a higher risk for developing insulin resistant. Whereas, in obese patients with low AT expression of IL-2, < 6.7-fold change; a non-significant association was found between IL-2 gene expression and FBG or HbA1c.

**Table 6:** Correlation of IL-2 low and high expression clusters observed in obese individuals with various clinical and biochemical markers.

Clinical parameter	Pearson correlation				
	IL-2 < 6.7-folds		IL-2 > 6.7-folds		
	(r value, *n* = 12)	*P-*value	(r value, *n* = 12)	*P-*value	
PBF	0.118	0.716	− 0.236	0.437	
BMI	− 0.017	0.953	− 0.158	0.575	
CHOL	0.102	0.728	0.447	0.095	
HDL	− 0.247	0.396	0.049	0.862	
LDL	0.15	0.608	0.252	0.365	
TGL	0.121	0.681	0.472	0.076	
FBG	− 0.338	0.237	0.566*	**0.028**	
HbA1c	− 0.293	0.309	0.653**	**0.008**	

Bold is used to highlight the significant values

Next, in order to determine which inflammatory and clinical metabolic parameters independently correlated with high IL-2 transcripts in the AT; the parameters having significant association were included for multiple stepwise regression analysis. Multiple regression analysis indicated that HbA1c (β = 5.089; P = 0.010) and TLR10 (β = 0.102; P = 0.030) were independently associated with IL-2 > 6.7-fold change cluster of individuals (Table 7).

**Table 7:** Multi linear regression-non-diabetic obese individuals expressing low and high expression levels of IL-2. Dependent variable: IL-2.

ANOVA (Sig) R2 = 0.55; *p* < 0.003		
Predictor variable	Beta (β) value	*P-*value
HbA1c	5.089	0.01
TLR10	0.102	0.03

**Figure 6:**
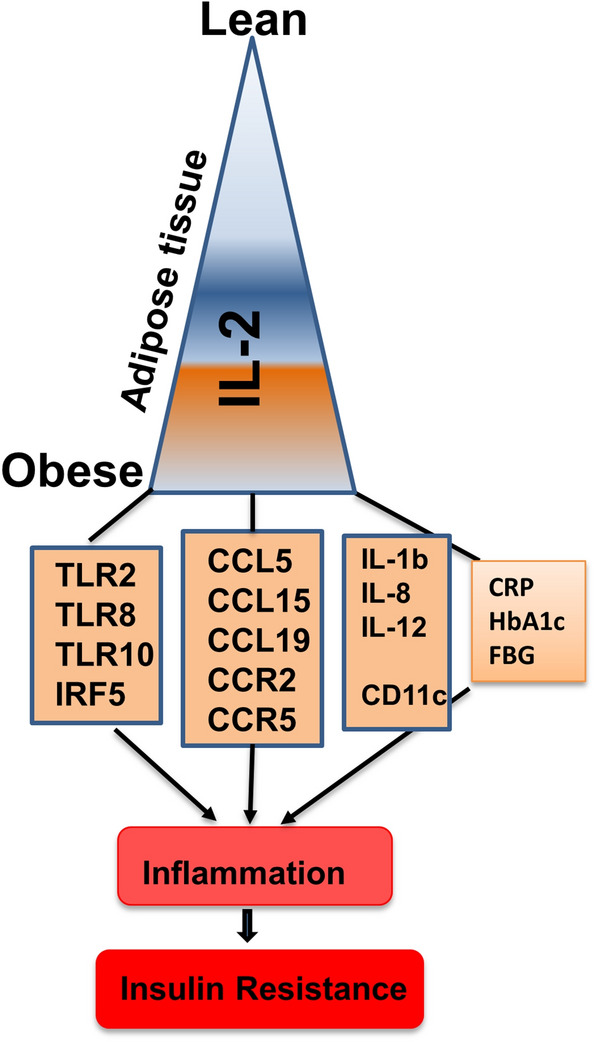
Increased adipose IL-2 expression corresponds with metabolic inflammation. The illustration displays a proposed model of metabolic inflammation, in support of the data presented, wherein the increase in obesity parallels with IL-2 adipose upregulation (positive relations with BMI and insulin resistance signatures; HbA1c and FBG). These alterations in the IL-2 expression are in agreement with various markers of adipose inflammation including inflammatory cytokines/chemokines, TLRs and TLR-associated signaling molecule IRF5, inflammatory macrophage marker (CD11c), as well as systemic immune-metabolic marker i.e., a positive association with circulatory CRP levels. Taken together, these changes support the IL-2 upregulation may be a marker for adipose tissue inflammation in obesity.

The thematic illustration supporting the afore-mentioned data is shown in Fig. 6.

## Discussion

Obesity-associated perturbations in inflammatory cytokines and chemokines are known to induce insulin resistance and lead to the development of T2D and its associated complications^28–32^. IL-2 is a proinflammatory cytokine, predominantly expressed by T-helper type 1 lymphocyte subset and it plays a key role in several inflammatory disorders such as multiple atherosclerosis^33^ and osteosclerotic myeloma^34^. In this study, we show that IL-2 gene and protein expression were significantly increased in the AT biopsies of obese individuals compared to their lean counterparts and these changes correlated with BM. In obesity, IL-2 is produced from the activated T lymphocytes that infiltrate the AT^15^, which may explain the observed IL-2 transcripts elevations in obese AT samples .

The white AT is a site for excessive energy storage and is also an active endocrine organ that secretes adipocytokines^35^. Next, we sought to determine how the changes in the AT expression of IL-2 mRNA related with the local gene expression of important inflammatory mediators. Utilizing RNA extracted from AT isolated from overweight and obese individuals, we found that transcript/protein levels of IL-8, IL-12A, CCL5, CCL19, CCR2 and CCR5 correlated with IL-2 mRNA/protein levels in the AT. Thus, it implies that obesity could be a predictor of AT dysfunction, associated with infiltration of monocytes/macrophages and activated T-cells as the source of IL-2. Relative to lean individuals, levels of the described interleukins were higher in obese individuals, particularly, in those with elevated levels of IL-2. In the AT, cytokines are secreted by adipocytes, monocytes and macrophages at different levels. They are also implicated in the pathogenesis of various inflammatory disorders such as atherosclerosis, coronary arty disease and diabetes^36^. In the context of obesity, our results imply that the co-expression of IL-2 with the studied inflammatory cytokines/chemokines could contribute to enhancing metabolic inflammation and insulin resistance in the AT. Our multivariate analysis showed that IL-8 and IL-12A are independently associated with IL‐2 expression which suggests that the later may play a significant role in setting conditions that could favor the production of former cytokines. Simultaneous expression of TNF-α and IL-8 in the AT was found to be associated with macrophage infiltration into the fat^37^. A previous study showed that elevated expression of IL-12 associated with metabolic inflammation and insulin resistance; and IL-12 expression increased with the level of adiposity^38^. Our study supports the notion that biologically relevant changes in the IL-8 and IL-12 gene expression in AT might be linked with the increased IL-2 expression in obesity settings.

In obesity and during metabolic inflammation, C–C chemokines mediate the infiltration of leukocytes into the AT. Hence, elevated levels of several chemokines were observed in the AT from obese and/or insulin-resistant patients^39, 40^. In AT from obese individuals, our study revealed a significant positive correlation between IL-2 mRNA and those of CCL5 and CCL19 as well as chemokine receptors including CCR2 and CCR5, all of which have been associated with obesity and metabolic inflammation^41^, and macrophage recruitment to AT^42^ . Notably, IL-2 gene expression in the AT from overweight and obese individuals correlated positively with that of IL-8 and IL-12A, but not with the closely related proinflammatory cytokines TNF-α or IL-6, indicating a non-randomized type of association between IL-2 and other proinflammatory mediators.

In state of obesity, insulin resistance is generally attributed to chronic low-grade systemic inflammation as well as AT inflammation, due in part to, increased production of inflammatory mediators^35, 43, 44^, causing the release of free fatty acids from lipolysis^19^. The binding of free fatty acids to TLRs on the surface of adipocytes and marcrophages generates an activation loop with prompt release of cytokines, explaing the obeseved positive correlation between IL-2 and members of TLR family, such as TLR-2, -8 and -10. It alludes to a functional role of IL-2 as a proimflamatory mediator. TLRs play an essential role in sensing the pathogens and shaping the innate immunity during the early stages of infection. TLR signaling activates the adaptor proteins, protein kinases and downstream effector molecules/transcription factors such as NF-κB and interferon regulatory factors (IRFs), leading to the expression of type-I interferons, TNF-α, and other inflammatory cytokines. TLRs’ role in metabolic inflammation and insulin resistance is well documented. TLR2, TLR4, TLR8 and TLR10 are higly expressed in the obese adipose tissue compared to lean. These TLRs have been involved in metabolic inflammation and insulin resistance^32, 45–47^. Our data show that TLR2, TLR8 and TLR10 correlate with IL-2 gene/protein expression in the AT, suggesting that TLRs play a significant role in setting a state which favors the increased expression of IL-2.

In overweight and obese individuals, IL-2 expression levels correlated significantly with CRP, implying that IL-2 is linked to systematic inflammation. CRP correlated with cellular activation and production of chemokines^48^ and independently associated with HOMA‐IR^49^. Moreover, AT IL-2 transcripts correlated positively with the plasma TGL levels, which was in agreement with the previously observed significant correlation between TGL and obesity^50^. Elevated TGL was found to be a risk factor for cardiovascular disease, independently of the high‐density lipoprotein cholesterol levels^51^. Hypertriglyceridemia with low high‐density lipoprotein cholesterol is regarded as a key feature of hypertension and metabolic syndrome in obese individuals^52^. Furthermore, our data show a significant positive association of IL-2 expression in the AT with insulin resistance signatures including FBG and HbA1c in overweight and obese individuals. Interestingly, expression of IL-2 in the AT from obese individuals revealed two distinct expression patterns (high and low, Fig. 4). Obese individuals with elevated levels of IL-2 gene expression in the AT showed a strong positive correlation with FBG and HbA1c which implies that increased IL-2 expression might be linked to hyperglycemia and/or insulin resistance. Notably, significant elevations in IL-2 expression in the AT together with that of other inflammatory mediators (IL-8, IL-12A, CCL5, CCL19, CCR2 and CCR5 and TLRs) in obesity suggests that these factors may contribute cooperatively for insulin resistance induction in this population.

In conclusion, our data show that AT IL-2 gene/protein expression was increased in obesity and it positively correlated with metabolic and inflammatory signatures, along with insulin resistance markers. Thus, IL-2 may represent as a novel biomarker for progression of metabolic inflammation and insulin-resistance.

## Material and methods

### Study population and anthropometric measurements

The study cohort comprises of 56 non-diabetic individuals, including 9 lean, 18 overweight and 29 obese, based on their body mass index (BMI i.e. ratio of body weight (kg) to height (m^2^). In accordance with the ethical guidelines of the Declaration of Helsinki which is approved by the ethics committee of Dasman Diabetes Institute, Kuwait, (RA 2010-003); each study participant submitted a written informed consent for inclusion in the study. The body waist circumference was measured using constant tension tape; height and weight were measured using calibrated portable electronic weighing scales and portable inflexible height measuring bars, respectively. IOI353 Body Composition Analyzer (Jawon Medical, South Korea) was used to examine the whole-body composition, percent body fat, soft lean mass and total body water.

### Collection of subcutaneous adipose tissue

Human AT biopsies (about 0.5 g) were collected from the abdominal subcutaneous fat pad just lateral to the umbilicus using standard sterile surgical methods. Briefly, periumbilical area was swabbed with ethanol and then locally anesthetized with 2% lidocaine (2 ml, Fresenius Kabi, LLC., Lake Zurich, IL, USA). Through a small superficial skin incision (0.5 cm), fat tissue was collected. After removal, biopsy tissue was further incised into smaller pieces (~ 50–100 mg) and preserved in RNAlater (Sigma-Aldrich Chemie GmbH, Taufkirchen, Germany) and stored at − 80 °C until use^53^.

### Measurement of metabolic inflammatory markers

Peripheral blood was collected from overnight-fasted individuals and analyzed for FBG, lipid profile, HbA1c, fasting insulin and C-reactive protein (CRP). Glucose and lipid profiles including plasma triglycerides (TGL), low-density lipoproteins (LDL), high-density lipoproteins (HDL), and cholesterol levels were measured using Siemens Dimension RXL chemistry analyzer (Diamond Diagnostics, Holliston, MA, USA)^47, 54, 55^. HbA1c was measured using Variant device (BioRad, Hercules, CA, USA). Homeostatic Model Assessment of Insulin Resistance (HOMA-IR) as a measure of insulin resistance was calculated from basal (fasting) glucose and insulin concentrations using the following standard formula: HOMA-IR = fasting insulin (μU/L) × fasting blood glucose (mmol/L) / 22.5. Plasma high sensitivity (hs) CRP levels were measured by ELISA (Biovendor LLC, Asheville, NC, USA). All assays were performed following instructions of the manufacturers.

### Real-time quantitative reverse transcription-polymerase chain reaction (qRT-PCR)

Total RNA was extracted from AT using RNeasy kit (Qiagen, Valencia, CA., USA) following the manufacturer’s protocol. The first strand cDNA was synthesized from 0.5 µg RNA using High Capacity cDNA Reverse Transcription kit (Applied Biosystems, CA, USA) as previously described^40, 56^. Real-time qRT-PCR was performed as we described previously^40, 57^; cDNA samples (50 ng) were amplified using TaqMan Gene Expression Master Mix (Applied Biosystems, CA, USA) and gene-specific 20 × TaqMan gene expression assays (Supplementary Table S2, Applied Biosystems, CA, USA) containing forward and reverse primers and target-specific TaqMan MGB probe labeled with FAM dye at the 5′-end and NFQ-MGB at the 3′-end of the probe using 7500 Fast Real-Time PCR System (Applied Biosystems, CA, USA). Each cycle involved denaturation (15 s at 95 °C), annealing/extension (1 min at 60 °C) after UDG (2 min at 50 °C) and AmpliTaq gold enzyme (10 min at 95 °C) activation. Relative gene expression to control, lean AT, was calculated using comparative Ct method as we previously described^56, 58, 59^. Results were normalized to GAPDH, and averages ± standard error of the mean (SEM) are shown relative to controls as indicated^60^.

### Immunohistochemistry (IHC)

Paraffin-embedded sections (4 μm thick) of subcutaneous adipose tissue were deparaffinized in xylene and rehydrated through descending grades of ethanol (100, 95, and 75%) to water. Antigen retrieval was performed by placing slides in target retrieval solution (pH 6.0; Dako, Glostrup, Denmark) in the pressure cooker boiling for 8 min and cooling for 15 min. After washing in PBS, endogenous peroxidase activity was blocked with 3% H_2_O_2_ for 30 min and non-specific antibody binding was blocked with 5% nonfat milk for 1 h followed by 1% bovine serum albumin solution for 1 h. The slides were incubated at room temperature overnight with primary antibodies against IL-2 (abcam ab180780, Cambridge, MA, USA: (1:200); IL-8 ( abcam, ab106350; 1:200 dilution pH6), CCL5 ( abcam, ab9679; 1:200 pH6), CD11c ( abcam, ab52632;1:300 pH6), TLR10 ( abcam, ab115598; 1:600 pH6), IRF5 ( abcam, ab140593; 1:400 pH6). After washing with PBS (0.5% Tween), slides were incubated for 1 h with secondary antibody (goat anti-rabbit conjugated with horseradish peroxidase (HRP) polymer chain; EnVision Kit from Dako, Glostrup, Denmark) and color was developed using 3,3ʹ-diaminobenzidine (DAB) chromogen substrate. Specimens were washed in running tap water, lightly counterstained with Harris hematoxylin, dehydrated through ascending grades of ethanol (75, 95, and 100%), cleared in xylene, and finally mounted in dibutylphthalate xylene (DPX). For analysis, digital photomicrographs of the entire adipose tissue sections (20×; PanoramicScan II, 3DHistech, Hungry. URL: https://www.3dhistech.com/products-and-software/hardware/pannoramic-digital-slide-scanners/pannoramic-scan-2/) were used to quantify the Immunohistochemical staining in three different regions to assess the regional heterogeneity in tissue samples and the regions were outlined using Aperio ImageScope software (Aperio Vista, CA, USA. URL: https://aperio-imagescope.software.informer.com/9.0/). The Aperio-positive pixel count algorithm (version 9), integrated into the Imagescope Software, was used to quantify the intensity of specific staining in the region. The number of positive pixels was normalized to the number of total (positive and negative) pixels to account for variations in the size of the region sampled. Color and intensity thresholds were established to detect the immuno-staining as positive and background staining as negative pixels. Once the conditions were established, all slides were analyzed using the same parameters. The resulting color markup of the analysis was confirmed for each slide^59, 61^.

### Statistical analysis

Statistical analysis was performed using GraphPad Prism software (La Jolla, San Diego, CA, USA) and SPSS for Windows version 19.01 (IBM SPSS Inc., Chicago, IL, USA) as previously reported ^47, 62^ . Data are shown as mean ± standard deviation (SD) values, unless otherwise indicated. Unpaired Student t-test was used to compare means between groups. Correlation and stepwise multiple regression analysis were performed to determine association between different variables. For all analyses, P value < 0.05 was considered significant.

## Supplementary information

Supplementary Information.
