# Supplementary material for: Elevated adipose tissue associated IL-2 expression in obesity correlates with metabolic inflammation and insulin resistance
Source: Sci Rep. 2020 Oct 1;10:16364. doi: 10.1038/s41598-020-73347-y (PMC7530670; doi:10.1038/s41598-020-73347-y)
Supplement: Supplementary file 1 — Supplementary Information. [file 41598_2020_73347_MOESM1_ESM.pdf]

Title: **Elevated adipose tissue associated IL-2 expression in obesity correlates with metabolic inflammation and insulin resistance**

Authors: Shihab Kochumon[1#], Ashraf Al Madhoun[2,3#], Fatema Al-Rashed[1,4], Reeby Thomas[1], Sardar Sindhu[2], Ebaa Al-Ozairi[5], Fahd Al-Mulla[3], Rasheed Ahmad[1*]

Affiliation: [1]Immunology & Microbiology Department, Dasman Diabetes Institute, Kuwait [2]Animal and Imaging Core Facilities, Dasman Diabetes Institute, Kuwait

[3]Genetics and Bioinformatics, Dasman Diabetes Institute, Kuwait, Kuwait

[4]Immunology Department, Ministry of Health, Kuwait

[5]Medical Division, Dasman Diabetes Institute, Kuwait

*__Correspondence:__ Rasheed Ahmad, PhD. Immunology & Microbiology Department, Dasman Diabetes Institute, Al-Soor Street, Kuwait, P.O. Box 1180 Dasman, 15462 Kuwait. Phone: 965 2224 2999 Ext. 4311; Fax: 965 2249 2406; E-mail: rasheed.ahmad@dasmaninstitute.org

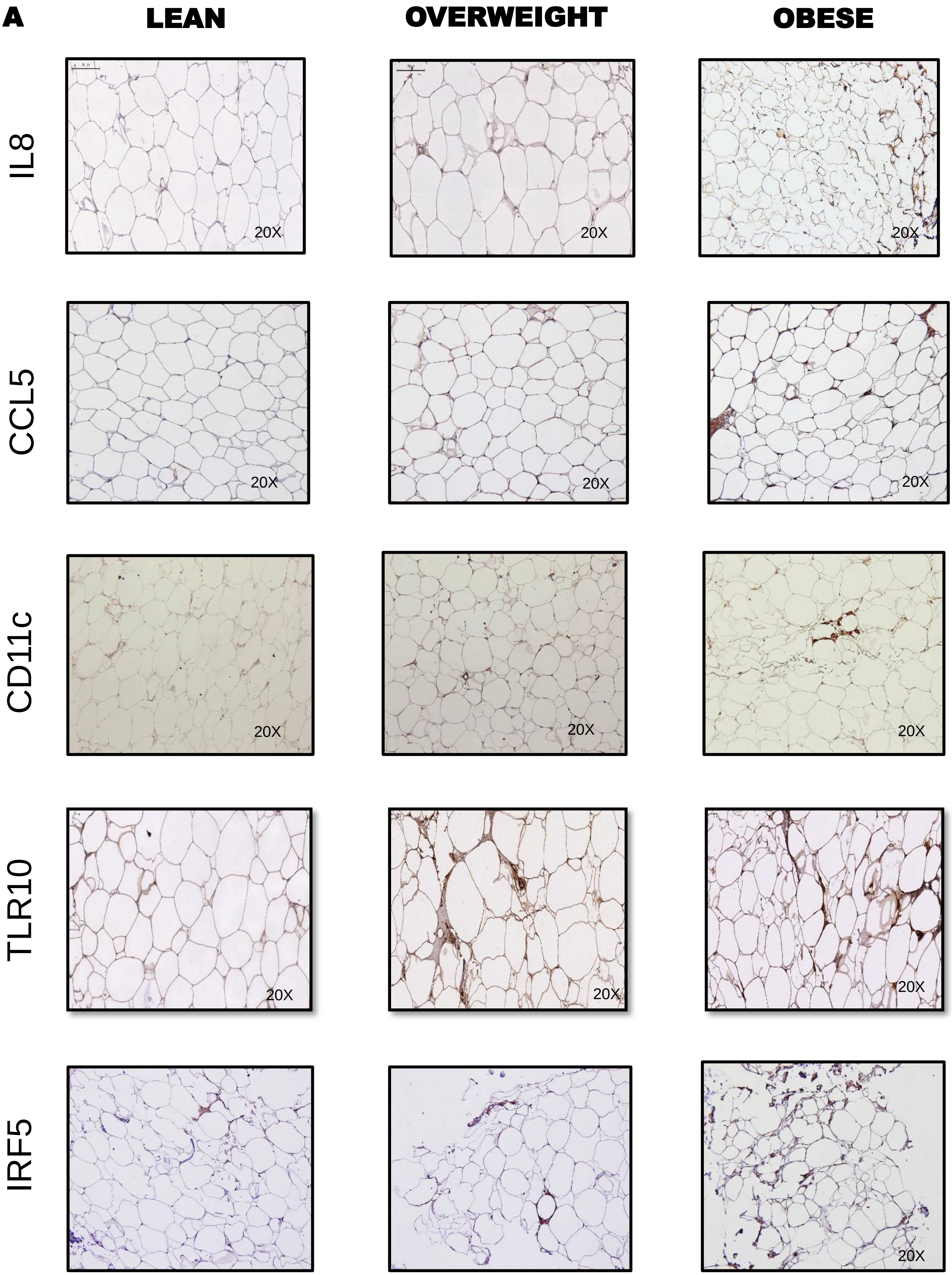
A
LEAN
OVERWEIGHT
OBESE
IL8
CCL5
CD11c
TLR10
IRF5
20X

B

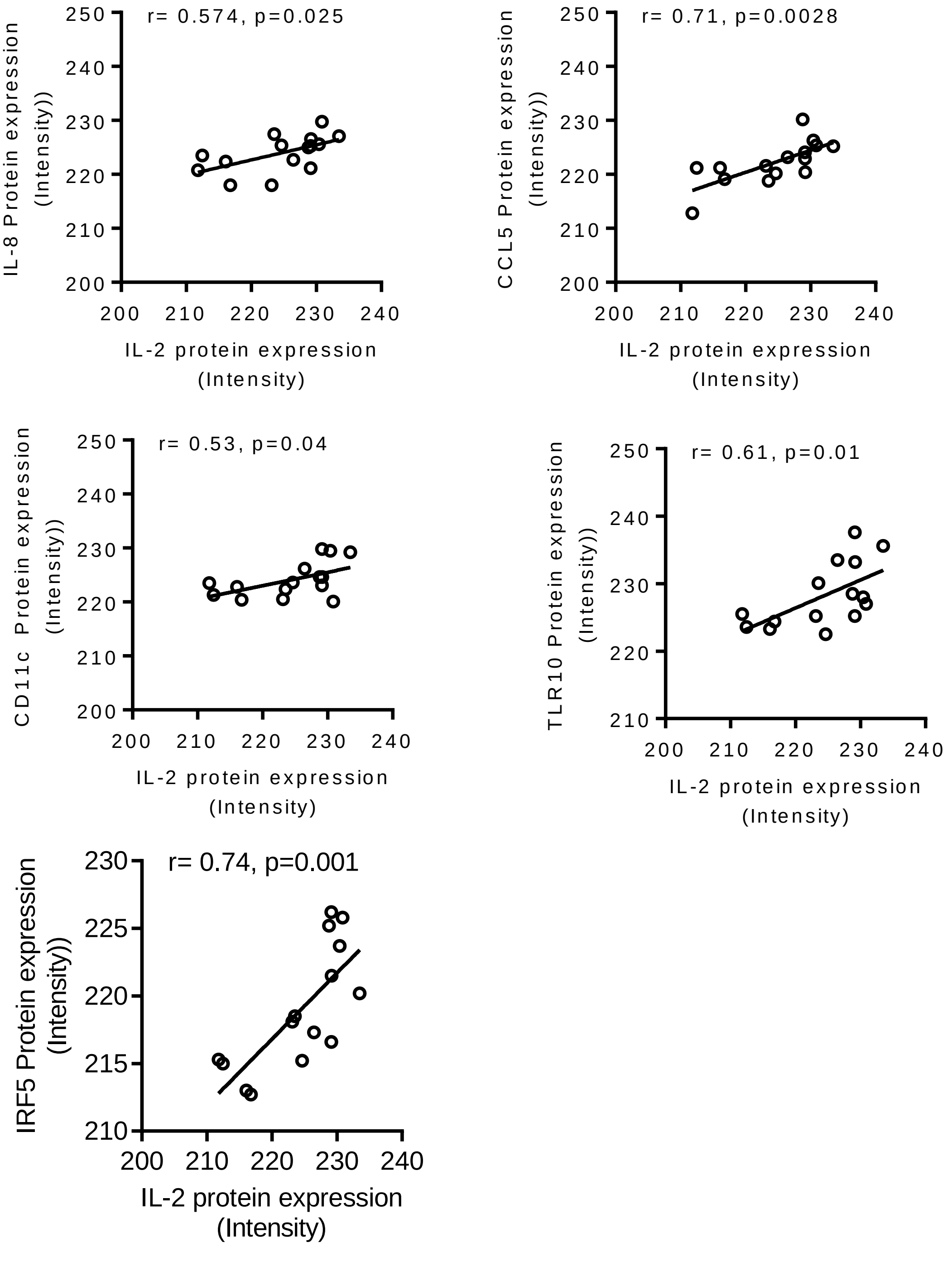

Figure S1. Increased protein expression of IL-8, CCL5, CD11c, TLR10 and IRF5 in adipose tissues and its correlation with IL-2. Protein expression of IL-8, CCL5, CD11c, TLR10 and IRF5 expression was determined in five lean, 5 overweight, and 5 obese non-diabetic individuals using immunohistochemistry (IHC) as described in Materials and Methods. (**A**) The representative IHC images obtained from three independent determinations with similar results show increased adipose tissue IL-8, CCL5, CD11c, TLR10 and IRF5 expression (20X magnification) in overweight and obese individuals compared with lean. (**B**) Staining intensity was determined based on Aperio-positive pixel counts (Aperio software algorithm version 9.0). IL-2 protein was expressed as IHC intensity in arbitrary units correlated with protein expression of IL-8, CCL5, CD11c, TLR10 and IRF5. All p-values ≤ 0.05 were considered as statistically significant.

Table S1: Partial Correlation of IL-2 with other markers adjusted for <u>High/Low IL-2 cluster</u> variances

control Variable	Variable	r-value	p-value
High/Low IL-2 Clusters			
	IL8	0.406	0.004
	IL12A	0.168	0.287
	CCL5	0.572	<0.0001
	CCL19	0.582	<0.0001
	CCR2	0.599	<0.0001
	CCR5	0.561	<0.0001
	ITGAX	0.279	0.043
	CD163	0.116	0.395
	TLR2	0.323	0.027
	TLR8	0.269	0.049
	TLR10	0.366	0.007
	IRF5	0.263	0.063

To validate the possibility of significant associations seen between IL-2 and cytokines/chemokines in Lean/OW/obese subjects could be because of the variances in High/Low IL-2 clusters, Partial Correlation was performed with IL-2 and other markers while controlling for the effect of High/Low IL-2 variances. After adjustment for IL-2 clusters variances all the cytokine/chemokine markers remain significantly correlated except with IL-12A and CD163.

Table S2: List of TaqMan Assays used for qRT-PCR

Primer List	Assay ID	Primer List	Assay ID
IL-1β	Hs01555410_m1	ITGAX/CD11c	Hs00174217_m1
IL-5	Hs01548712_g1	CD163	Hs00174705_m1
IL-6	Hs00985639_m1	CXCL9	Hs00171065_m1
IL-8	Hs00174103_m1	CXCL10	Hs01124251_g1
IL-10	Hs00961622_m1	CXCL11	Hs04187682_g1
IL-12A	Hs01073447_m1	TNFα	Hs01113624_g1
IL-13	Hs00174379_m1	TGFβ	Hs00820148_g1
IL-18	Hs01038788_m1	IFNB1	Hs01077958_s1
CCL2	Hs00234140_m1	TLR2	Hs01872448_s1
CCL3	Hs04194942_s1	TLR3	Hs01551078_m1
CCL5	Hs00982282_m1	TLR4	Hs00152939_m1
CCL8	Hs04187715_m1	TLR7	Hs01933259_s1
CCL11	Hs00237013_m1	TLR8	Hs00152972_m1
CCL15	Hs00361122_m1	TLR9	Hs00370913_s1
CCL19	Hs00171149_m1	TLR10	Hs01935337_s1
CCL20	Hs01011368_m1	MyD88	Hs01573837_g1
CCR1	Hs00928897_s1	IRAK1	Hs01018347_m1
CCR2	Hs00704702_s1	TRAF6	Hs00371512_g1
CCR5	Hs99999149_s1	IRF5	Hs00158114_m1
CD16	Hs04334165_m1	GAPDH	Hs03929097_g1
